# Repurposing of Benzimidazole Anthelmintic Drugs as Cancer Therapeutics

**DOI:** 10.3390/cancers14194601

**Published:** 2022-09-22

**Authors:** Bomi Song, Eun Young Park, Kwang Joon Kim, Sung Hwan Ki

**Affiliations:** 1 Graduate School of Clinical Pharmacy, Chosun University, Gwangju 61452, Korea; 2 College of Pharmacy, Mokpo National University, Mokpo 58554, Korea

**Keywords:** repurposing, benzimidazole, anthelmintic drugs, cancer therapy

## Abstract

**Simple Summary:**

Although non-prescription anthelmintics are often used for cancer treatment, there is a lack of information regarding their anti-cancer effects in clinical settings. The aims of our review are to describe the possibilities and limitations of the anti-cancer effects of benzimidazole anthelmintics and to suggest ways to overcome these limitations. The results of the current review illustrate the potential development of anthelmintics as a useful strategy for cancer treatment based on much preclinical evidence. Furthermore, they suggest that more rigorous studies on whole anti-cancer pathways and development strategies, including formulations, could result in significantly enhanced anti-cancer effects of benzimidazoles as a repurposed cancer therapy in clinical settings.

**Abstract:**

Benzimidazoles have shown significant promise for repurposing as a cancer therapy. The aims of this review are to investigate the possibilities and limitations of the anti-cancer effects of benzimidazole anthelmintics and to suggest ways to overcome these limitations. This review included studies on the anti-cancer effects of 11 benzimidazoles. Largely divided into three parts, i.e., preclinical anti-cancer effects, clinical anti-cancer effects, and pharmacokinetic properties, we examine the characteristics of each benzimidazole and attempt to elucidate its key properties. Although many studies have demonstrated the anti-cancer effects of benzimidazoles, there is limited evidence regarding their effects in clinical settings. This might be because the clinical trials conducted using benzimidazoles failed to restrict their participants with specific criteria including cancer entities, cancer stages, and genetic characteristics of the participants. In addition, these drugs have limitations including low bioavailability, which results in insufficient plasma concentration levels. Additional studies on whole anti-cancer pathways and development strategies, including formulations, could result significant enhancements of the anti-cancer effects of benzimidazoles in clinical situations.

## 1. Introduction

According to several cross-sectional surveys, more than one-third of cancer patients in Japan, Poland, and Wales receive unconventional therapies to support their cancer treatments or replace conventional therapies, [1,2,3]. This may be explained as follows. As shown in the statistics reported in studies by Jung et al. [4] and the American Cancer Society [5], the 5-year relative survival rates for cancer patients reported between 1993 and 1995 in South Korea and between 1995 and 1997 in the United States were 41.5% and 63%, respectively. Meanwhile, in South Korea between 2012 and 2016 and in the United States between 2011 and 2017, the rates have been revealed to be 70.6% and 68%, respectively. Even though this is a sharp improvement, the rates remain low.

First, cancer remains a fatal disease that ranks high as a cause of death globally, despite several decades of efforts to develop medicines for its treatment [6]. Even though various new approaches including targeted drug therapy (e.g., erlotinib, atezolizumab, and bevacizumab) have recently been introduced, the survival rates of patients with pancreatic cancer or liver cancer are still reported to be less than 70% [7,8]. Therefore, many cancer patients are still in dire need of alternative medicines for cancer treatment.

Second, the development of new anti-cancer drugs is becoming increasingly difficult. Many pharmaceutical companies faced several challenges in the 2000s, such as patent cliffs and intense generic competition, in addition to a stagnating success rate for new drug approval by the Food and Drug Administration (FDA) due to cost increases and strengthened approval requirements [9,10]. A recent study estimated that the mean research and development cost for a new drug is $985.3 million, with the cost for anti-cancer drugs reaching $2771.6 million [11].

Third, many cancer patients are likely to be under financial pressure due to the high cost of cancer treatment. According to Iragorri et al., the portion of the cost of cancer care paid for by patients could account for approximately 40% of their annual income in low- and middle-income countries [12]. Thus, the consumption of alternative drugs might be attributed to the fact that there are still limited remedies to effectively suppress various types of cancers and no complete and cost-effective medicine that offers a cure.

Therefore, repurposing drugs with anti-cancer efficacy can be considered an important strategy for cancer therapy. As part of repurposing drugs for cancer treatment, a number of medicines, including metformin, itraconazole, and indomethacin [13], which have been developed or approved for other diseases, have been attracting interest. Among other drugs, benzimidazoles have shown significant promise, with various studies revealing their anti-cancer effects and relatively safe properties over long periods of use [14]. In addition, they have also attracted public attention in South Korea owing to a talk by Joe Tippens describing a complete recovery from his lung cancer after using benzimidazole [15].

**Figure 1:**
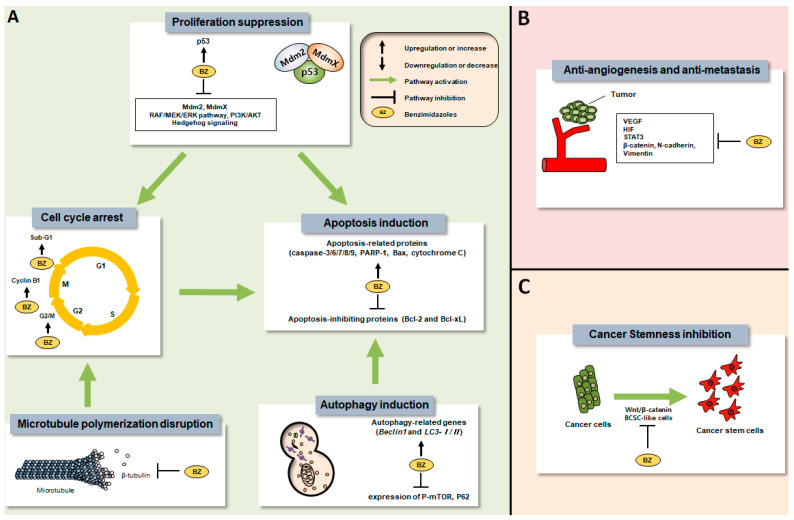
Schematic diagram of anti-cancer mechanisms of benzimidazoles. (**A**) Effects of benzimidazoles on cancer cell death, (**B**) Anti-angiogenic and anti-metastatic effects of benzimidazoles, (**C**) Inhibitory effects of benzimidazoles on cancer stemness. Abbreviations: Mdm2: mouse double minute 2 homolog; MdmX: mouse double minute 4; RAF: rapidly accelerated fibrosarcoma; MEK: mitogen-activated protein kinase; ERK: extracellular signal-regulated kinases; PI3K: phosphatidylinositol 3-kinase; AKT: protein kinase B; LC3: microtubule-associated protein 1 light chain 3; mTOR: mammalian target of rapamycin; PARP: poly(ADP-ribose) polymerase; Bax: B-cell lymphoma 2 (Bcl-2)-associated X protein; Bcl-2: B-cell lymphoma 2; Bcl-xL: B-cell lymphoma extra-large; VEGF: vascular endothelial growth factor; HIF: hypoxia-inducible factor; STAT3: signal transducer and activator of transcription 3; BCSC: breast cancer stem cell.

Several chemical groups are classified as anthelmintic drugs, including benzimidazoles (e.g., albendazole), halogenated salicylanilides (e.g., niclosamide), imidazothiazole derivatives (e.g., levamisole), thiazolides (e.g., nitazoxanide), macrocyclic lactones (e.g., ivermectin), antitrematodals (e.g., praziquantel), quinolines (e.g., pyrvinium), and piperazine [16]. This review focuses on the anti-cancer activities of benzimidazoles and includes the following 11 drugs (Table 1): albendazole (ABZ), fenbendazole (FBZ), flubendazole (FLZ), mebendazole (MBZ), carbendazim (CBZ), methiazole (MTZ), nocodazole (NCZ), oxfendazole (OFZ), oxibendazole (OBZ), ricobendazole (RBZ), and parbendazole (PBZ). Among these, ABZ and MBZ have been approved by the FDA for fighting parasitic infections in humans [17], while FBZ, OFZ, and OBZ have been approved for veterinary parasite treatment [17]. Like other microtubule-targeting agents that have been widely used for cancer treatment [18], benzimidazole anthelmintics, which exert anti-parasitic effects by inhibiting microtubule polymerization [19,20], have also been regarded as having anti-cancer effects, and in practice, the chemical group has exhibited tumor suppression in many studies. Various research studies have revealed that the anti-cancer activities of benzimidazoles can be attributed to underlying mechanisms such as the disruption of microtubule polymerization [21,22,23,24,25,26], the induction of apoptosis [19,26,27,28,29,30,31], or the inhibition of angiogenesis [32,33], metastasis [19,26,33,34], etc. The anti-cancer mechanisms of benzimidazoles are illustrated in Figure 1.

This review aims to elucidate the comprehensive anti-cancer efficacies of benzimidazole anthelmintics in terms of the in vitro, in vivo, and clinical evidence available to date, and to summarize their pharmacokinetic properties.

## 2. Preclinical Anti-Tumor Efficacies of Benzimidazole Anthelmintics

### 2.1. ABZ

#### 2.1.1. In Vitro Anti-Tumor Effects

ABZ offers various possibilities for use in cancer therapies, with diverse advantages beyond its effects in microtubule inhibition, such as its ability to suppress growth of a wide range of cancer cells, for example, those of the brain [20], breast [19,35], colon [36], stomach [21], leukemia [37], liver [38], lung [39], ovary [40], and skin [22]. It is noteworthy that ABZ exerted cytotoxicity against a human glioblastoma multiforme stem-like neurosphere cell line at a low half maximal inhibitory concentration (IC_50_) of 0.1 μM [20]. As drug delivery through the blood-brain barrier (BBB) is critical in the treatment of brain cancer, the fact that ABZ has been used as a medicine for central nervous system parasitic infections, because of its physicochemical ability to penetrate the BBB [20], suggests its potential for brain cancer therapy. Triple-negative breast cancer (TNBC) and Kirsten rat sarcoma virus (KRAS)-mutant lung cancer have limited therapeutic options, but ABZ has been reported to suppress TNBC- [19,35] and KRAS-mutant [39] cells. In addition, ABZ inhibits VEGF secretion in ovarian cancer cells [41].

Moreover, the use of ABZ in combination with radiation resulted in a synergistic increase in the sensitivity of lung (H153 and H446) and skin (A375 and A2058) cancer cells to radiation [42,43]. ABZ enhanced radiation-induced apoptosis by a dual effect of cell cycle arrest in the G2/M phase and induction of DNA damage, while paclitaxel sensitizes melanoma to radiation only by G2/M cell cycle arrest [42].

#### 2.1.2. In Vivo Anti-Tumor Effects

In line with the in vitro results, ABZ showed in vivo inhibitory effects in brain [44], breast [19,35], lung [32], and ovarian [41] cancer. There is evidence that ABZ has in vivo anti-tumor effects in glioma (GL261 syngeneic mouse model) [20], non-small cell lung cancer (NSCLC) (A549 xenografts) [32], and TNBC cells (MDA-MB-231 xenografts and orthotopically injected 4T1 cells) [19,35]. In addition, ascites formation is known to be associated with mortality in patients with peritoneal cancers caused by several cancer types, including ovarian cancer [41]. Interestingly, ABZ did not show distinct anti-tumor effects in ovarian cancers, but reduced ascites formation by inhibiting VEGF expression, and thus, eventually inhibiting angiogenesis [41,45,46]. Many in vivo tests have been conducted using mice, where the doses of ABZ were 30–300 mg/kg via oral administration (p.o.) [35,38], 1.5–450 mg/kg via intraperitoneal (i.p.) injection [46,47], and 1.5 mg/kg through tail vein injection [44]. A dose of 50 mg/kg via i.p. injection [48,49] was the most frequently used in the tests.

### 2.2. FBZ

#### 2.2.1. In Vitro Anti-Tumor Effects

FBZ has been reported to suppress cancer cells of the brain [50], breast [22], colorectal [51], lung [39,52], pancreatic [26], and skin [22]. Like ABZ, it has been used to treat intracranial parasites in dogs [50]; thus, it suggests utility in treating brain cancer owing to its BBB-penetrating characteristics [50]. In the case of lung cancer, Shimomura et al. observed that FBZ suppressed KRAS-mutant lung cancer cells to a greater extent than other benzimidazole derivatives by suppressing RAS-related signaling pathways [39]. As effective medicines have not yet been developed for mutant KRAS, this finding might provide a valuable option for treating lung cancer with mutant KRAS. In addition, FBZ showed enhanced apoptosis when it was tested in H460 and A549 human NSCLC cell lines having wild-type p53, compared to those (H522) with mutant p53, thus suggesting its important role in FBZ-induced apoptosis [52].

With regard to its use in combination with other medicines, FBZ did not show any effect on cellular radiosensitivity in EMT6 mouse mammary tumor cells, even though it shares a similar chemical structure to that of benzimidazoles having an effect as hypoxic cell radiosensitizers [53], such as ABZ, which shows radiosensitizing activity in lung and skin cancer cells [42,43].

#### 2.2.2. In Vivo Anti-Tumor Effects

Although the inhibitory effects of FBZ in cancer cells have been reported for several types of cancers in in vitro tests, its cancer inhibitory effects in vivo have been reported only in the case of lung cancer (A549 adenocarcinoma cell xenografts) in mice. When it was administered p.o. at a dose of 1 mg/mouse every other day for 12 days, tumor growth and vascularity were reduced and apoptosis was induced in tumor cells [52]. The reason that there have been few studies in vivo might be attributed to the restricted possibilities of FBZ for humans, owing to its regulatory approval only for veterinary application by the FDA. Thus, more studies are required to understand its anti-cancer effects.

### 2.3. FLZ

#### 2.3.1. In Vitro Anti-Tumor Effects

Among benzimidazole derivatives, FLZ has shown a suppressive effect on cell viability in a broad spectrum of cancer cell lines. In a study conducted by Michaelis et al., FLZ was evaluated for its inhibitory effect on 321 cell lines of various cancer types [54]. In that screening study, FLZ showed a remarkable tumor cell inhibition effect on three kinds of cancers, i.e., multiple myeloma, neuroblastoma, and leukemia/lymphoma, while the effect on the other cancers was more modest. The study focused on the anti-cancer effect against neuroblastoma, and one of the main mechanisms was explained in relation to p53-mediated apoptosis, which has been demonstrated to play an important role during tumor cell inhibition of FLZ in UKF-NB-3 neuroblastoma cells in that study.

Notably, it has shown potential for use in breast cancer therapy. First, it is difficult to treat TNBC, because sufficient targeted therapies have not yet been found; however, FLZ elicited anti-tumor effects in TNBC by suppressing cell migration [28,55], inducing autophagy [56,57], and influencing a number of mechanisms that inhibit breast cancer stem cell (BCSC)-like properties [28,55] which are related to metastasis, recurrence, and drug resistance. Second, FLZ treatment significantly downregulated human epidermal growth factor receptor (HER)-2-related signaling in HER2-positive breast cancer and induced apoptosis in trastuzumab-resistant cell lines as well as in sensitive cell lines [58], which indicates its potential for use as a substitute or supplement for trastuzumab. Third, FLZ also suppressed BCSC-like properties in non-TNBC [55,58]. Thus, these findings warrant further investigations on the application of FLZ for breast cancer treatment.

FLZ administration can enhance anti-cancer effects through a combination of several approved anti-cancer medicines. FLZ combined with conventional chemotherapy drugs, e.g., fluorouracil or doxorubicin, exerted a more enhanced cytotoxic effect in both cell viability and colony formation tests in breast cancer cells (MDA-MB-231 and BT-549) [55]. Furthermore, Spagnuolo et al. observed that FLZ inhibited tubulin polymerization, similar to vinblastine, but bound to a different binding site from that of vinblastine, thereby showing a cytotoxic effect in synergism with vinblastine in an OCI-AML2 leukemia cell line; in addition, cells that were resistant to vinblastine were suppressed by FLZ [59].

#### 2.3.2. In Vivo Anti-Tumor Effects

Some cancer cells in the brain [54], breast [28], colorectal [60], hematological [59], and skin [61] are susceptible to FLZ in vivo. Among in vivo studies, one used the chick chorioallantoic membrane assay [54], while most used mice as test animals, which were administered doses of FLZ ranging from 10 mg/kg to 200 mg/kg i.p [28,59,60,61]. In concordance with the in vitro results, FLZ showed a remarkable anti-tumor effect on neuroblastoma [54] via the inhibition of tumor growth and vessel formation in a chorioallantoic membrane assay for brain cancer cells (neuroblastoma xenograft) [54]. Furthermore, consistent with the in vitro results, it was also observed that FLZ can be effective for TNBC [28,55,57], showing delayed tumor growth or anti-migration activity, such as a decrease in matrix metalloproteinase-2, and trastuzumab-resistant xenografts in HER2-positive breast cancer [58]. With respect to combination therapy, one study showed that the use of FLZ in combination with vinblastine or vincristine caused more effective suppression, as compared to the administration of either drug alone, in a leukemia xenograft test [59].

### 2.4. MBZ

#### 2.4.1. In Vitro Anti-Tumor Effects

Similar to FLZ, MBZ showed extensive inhibitory effects on a wide range of cancer cell lines. It has also been suggested that MBZ may be a useful therapy for brain cancer, based on its BBB-penetrating characteristics [20,50,62,63,64,65]. It was observed that MBZ could efficiently reduce BCSC-like cells in TNBC and interfere with the reprogramming of breast cancer cells into BCSCs, which are known to be induced after radiation therapy [66]. In head and neck squamous cell carcinoma (HNSCC) and acute myeloid leukemia (AML) [67], which are both aggressive types of cancers, MBZ showed a potent inhibitory effect. Notably, the anti-tumor effect of MBZ on HNSCC (CAL27 and SCC15) was more potent than that of cisplatin [68]. The proliferation of cancer cells was prominently suppressed at lower concentrations of MBZ than those of cisplatin in both HNSCC cell lines. The anti-tumor effects of MBZ are also related to inhibition of drug resistance. MBZ downregulated the expression of multiple drug resistance (MDR) genes (*ABCB1*, *ABCC1*, and *SLC47A1*) in malignant ascites cells [69]. In T cell acute lymphoblastic leukemia (T-ALL), MBZ was effective in suppressing the growth of cancer cell lines despite their chemoresistance, as shown in the test results that it inhibited camptothecin-resistant and MDR-1-overexpressing CEM/C1 cells [70]. Based on these activities, MBZ may be a potential adjuvant therapy for conventional anti-cancer treatments, to prevent drug efflux.

MBZ has been identified as a leading anti-cancer compound by screening established libraries in several studies. In a study conducted by Tan et al., upon screening 1448 molecules using comparative modeling studies, MBZ was discovered to be a TRAF2- and NCK-interacting kinase (TNIK) inhibitor. Since TNIK activates the Wnt/β-catenin/T-cell factor 4 pathway and its activation contributes to the transformation of cells to cancer cells, particularly colorectal cancer, it can be applied to Wnt-activated colorectal cancer [71]. In another study by Li et al., in which an in silico study was conducted, MBZ was also identified as one of the top 20 molecules inducing differentiation of HL-60 leukemia cells, upon analyzing gene expression profiles, including myeloid markers of leukemia cells, after exposure to 1235 molecules [72]. Moreover, in several screenings, MBZ showed a potent inhibitory effect on crucial kinases in both types of cancers, BRAF^WT^ and BRAF^V600E^ [73]. The dominant anti-tumor effects of MBZ, which were revealed in various screening tests, suggest its potential for various uses in anti-cancer therapy.

Finally, more studies have been performed on combination therapies of MBZ with conventional drugs than on any other benzimidazole compound. When MBZ was used with temozolomide, which is a standard therapy for glioblastoma multiforme, or with temozolomide and vinblastine as a triple combination, both combinations showed enhanced cytotoxicity compared to temozolomide alone [20,62]. Three studies reported that MBZ sensitized cancer cells to ionizing radiation through the inhibition of DNA damage response proteins in glioma cells [18] and the promotion of cancer cell apoptosis in meningioma [64] or TNBC cells [66].

#### 2.4.2. In Vivo Anti-Tumor Effects

Evidence of the tumor-suppressive effects of MBZ has also been found consistently in in vivo tests for challenging cancers such as brain cancer [20,63,64,65,74,75], TNBC [66], HNSCC [68], chemoresistant T-ALL [70], and AML [67]. In murine hepatocellular carcinoma, MBZ treatment resulted in outstanding effects, encompassing not only inhibition of tumor growth and angiogenesis, but also improved liver function and histology [76]. Moreover, MBZ revealed itself as a potential new strategy for chemoprevention in a familial adenomatous polyposis model using *APC^Min/+^* mice by reducing the number of polyps and tumor formation, which could contribute to suppressing the initiation of colorectal cancer [77]. Most of the in vivo tests were conducted using mice, where the doses of MBZ were 1–2 mg/mouse upon administration through p.o. [78,79], 25–100 mg/kg via p.o. [67,74], 7.5–100 mg/kg by means of i.p. injection [68,70], and 180 mg/kg by means of tail vein injection [80]. The most frequently selected administration was 50 mg/kg through p.o. [20,72,75]. It should be noted that MBZ was administered orally in most in vivo studies, whereas the other benzimidazoles, except OBZ, were administered as injections. Considering that the most used doses in the in vivo studies are decided based on previous in vivo results and in vitro data, it can be assumed that these doses and the use of the oral application in these in vivo tests were regarded as sufficient to reach the required concentrations for the anti-cancer effects of MBZ in vivo. Although data are limited, the reported bioavailability for MBZ in humans has been given as ‘5–10%’ and ‘17–22%’, i.e., higher than the reported bioavailability for ABZ in humans, which is ‘1–5%’ [81]. It is assumed that the higher bioavailability of MBZ than that of the other benzimidazoles might be the reason for its availability for oral application in vivo.

Combination therapies tested for MBZ in vivo are described below. When MBZ was used with radiation, enhanced inhibition of tumor growth was observed, compared to the use of radiation alone in TNBC [66] or MBZ alone in a rodent model of meningioma [64]. Finally, MBZ can also be used to develop new strategies for cancer therapy. The compound suggests a new modality for chemoprevention in a familial adenomatous polyposis model by improving the cancer-preventive effects when used in combination with sulindac (compared to those exhibited by sulindac alone), resulting in a reduction in the number and size of polyps and microadenoma formation through its anti-angiogenic activities and heightened anti-inflammatory effects [77]. This highlights the possibility of using this combination to prevent polyps from transforming into colorectal cancers. It seems that MBZ is in a better position for drug development than other benzimidazoles, owing to its advantages, such as relatively extensive preclinical studies and more useful application routes.

### 2.5. The Others

#### 2.5.1. In Vitro Anti-Tumor Effects

CBZ is a metabolite of benomyl that is used as a fungicide, unlike other benzimidazoles. Several studies have shown its anti-tumor activities against breast [25,82,83], colorectal [84], and liver [30] cancer cell lines; however, most of the studies on CBZ have been conducted in breast cancer cells. CBZ exerted more enhanced tumor cell inhibitory effects in MCF-7 breast cancer cells when it was used in combination with astaxanthin, a potent anti-oxidant, than when it was used alone, despite the controversy surrounding the combination of anti-oxidants with chemotherapeutics [82].

In the case of MTZ, one study showed its anti-tumor effects in lung cancer cells, where it was identified to be selectively effective against KRAS-mutant lung cancer cells, as compared to wild-type cells, in screening tests carried out using 1271 small molecules; the selectivity of MTZ was more obvious than that of other benzimidazoles [39]. When MTZ was used in combination with trametinib, a MEK inhibitor, a synergistic cytotoxic effect was observed in KRAS-mutant lung cancer cells [39].

To date, NCZ has shown inhibitory effects against two types of cancer cell lines: colorectal [84] and lung [24,39] cancer. Although the number of studies was insufficient, NCZ showed potent anti-tumor effects among the benzimidazoles in two studies. In a study on colorectal cancer cell lines (RKO and HCT-116), NCZ was shown to be one of the two compounds with the lowest IC_50_ values among the seven benzimidazoles tested [84]. In another study on NSCLC, the depolymerization of tubulin and abnormal spindle formation, which are assumed to be the key factors determining the progress of apoptosis, were greater with NCZ than with MBZ [24]. When NCZ was treated with an inhibitor of heat shock factor (HSF) 1 or the MEK/extracellular signal-regulated kinases (ERK) pathway, it exerted higher cytotoxicity than NCZ alone, thereby lessening the chemotherapeutic resistance promoted by ERK-1/2-dependent HSF1 in colorectal cancer cells [84].

Anti-tumor effects of OFZ in colorectal [84] and lung [85] cancer cells have been reported. In the A549 and H1299 NSCLC cell lines [85], OFZ inhibited cancer cell proliferation; this inhibitory effect was related to the suppression of c-Src signaling, which is known to mediate cell proliferation. OFZ repressed cancer cell viability against NSCLC cell lines more effectively in combination with cisplatin by enhancing the inhibition of c-Src activation and upregulation of p53 [85].

OBZ has shown anti-proliferative effects in lung [39], pancreatic [26], colorectal [51], skin [86], and prostate [31] cancers. Shimomura et al. showed that benzimidazole derivatives, including OBZ, suppressed KRAS-mutant lung cancer cells, but were not as effective as MTZ and FBZ [39]. In two types of pancreatic cancer cell lines (AsPC-1 and Capan-2), OBZ inhibited cell viability following PBZ, among the four benzimidazoles tested [26]. In a study conducted by Nygren et al., several compounds, including OBZ, which shared a benzimidazole pharmacophore were identified as one of several distinct clusters that were effective in suppressing tumor cell survival upon screening 1600 molecules in HCT 116 and RKO colorectal cancer cell lines [51]. In addition, OBZ was found to be one of the 10 compounds with tumor-inhibitory effects upon screening 2000 compounds against the M-14 and SK-Mel-19 melanoma cell lines [86]. Research on prostate cancer cells (22Rv1 and PC-3) showed that the anti-tumor mechanisms of OBZ increased the expression of two well-known tumor suppressors, microRNA (miRNA)-204 and p53 [31].

RBZ is a metabolite (albendazole sulfoxide) of ABZ that has shown anti-proliferative effects against a TNBC cell line (4T1) [35], as well as breast (MCF-7) [87], lung (NCI-H460) [87], and skin (A375-C5) [87] cancers; however, its effects were found to be milder than those of ABZ [35,87]. In addition, RBZ effectively suppressed colorectal cancer cells (HT-29) [36] but was not effective at any concentration when tested in four colon cancer cell lines (SW480, SW620, Caco2, and HCT8) [88]. Few studies have investigated the anti-tumor mechanisms of RBZ.

PBZ has shown anti-tumor effects in colorectal [84], lung [39], and pancreatic [26] cancer. In a study on colorectal cancer cell lines (RKO and HCT-116), PBZ was shown to be one of the two compounds with the lowest IC_50_ values, among a total of seven benzimidazoles tested [84]. Remarkably, it exerted the most potent cytotoxicity among the four benzimidazoles tested against pancreatic cancer [26]. In contrast, the anti-tumor effect of PBZ was not stronger than that of six other benzimidazoles tested, in the Z-score analysis for growth inhibition of KRAS-mutant and wild-type lung cancer cell lines, upon screening of 1271 compounds, where it was identified as one of 50 top-ranking compounds [39]. Similar to NCZ treatment, PBZ showed enhanced cytotoxicity and reduced chemotherapeutic resistance through ERK1/2-dependent HSF1 in colorectal cancer cells [84]. In addition, the inhibitory effect of PBZ was synergized when combined with gemcitabine, against pancreatic cancer cells (AsPC-1 and Capan-2) [26].

#### 2.5.2. In Vivo Anti-Tumor Effects

Only one study reported in vivo anti-tumor effects of the seven benzimidazoles, in which OBZ (25 mg/kg p.o., in mice) was shown to increase the expression levels of miRNA-204 and p53, in addition to exerting repressing effects on androgen receptors and prostate-specific androgens in prostate 22Rv1 tumors [31].

## 3. Clinical Properties of Benzimidazole Anthelmintics

### Clinical Evidence

Limited clinical evidence has been published for benzimidazole anthelmintics, with most of it being restricted to only three types of benzimidazoles: ABZ, CBZ, and MBZ (Table 2). For ABZ, one phase 1 clinical trial [89] and one pilot study [90] have been conducted. In both the studies, it was demonstrated that ABZ has modest anti-tumor effects, including reduction of tumor markers and a well-tolerated safety profile; however, dramatic effects such as complete recovery or survival prolongation have not been reported. For CBZ, one phase 1 trial (NCT00003709) has been completed, but the results cannot be found at ClinicalTrials.gov (https://clinicaltrials.gov/) (accessed on 1 December 2021) or in any research article. Lastly, two case reports have described the anti-cancer activities of MBZ. In these reports, which aimed to treat adrenal cancer [91] and metastatic colon cancer [92], metastases regressed without any significant adverse effects upon treatment with MBZ. In particular, a man with adrenocortical carcinoma showed stable disease status for 19 months during the application of MBZ. Meanwhile, although many clinical trials are being conducted on MBZ, most of them are scheduled to be completed after June 2022. The possible reasons for this large number of trials being conducted on MBZ might be its preclinical study history and dose convenience, since it has already been approved for human use by the FDA and can be applied orally because of its relatively higher bioavailability. Consistent with the preclinical study of its anti-tumor efficacy, owing to its BBB-penetrating ability [20,50,62,63,64,65], three of the eight trials dealt with brain tumors (NCT01729260, NCT02644291, and NCT01837862). Of note, one phase 2a trial (NCT03628079) conducted on 11 patients with advanced cancer of the gastrointestinal or unknown origin was terminated earlier than planned because of a lack of effect. Moreover, the six recent trials that are currently ongoing (NCT04443049, NCT01729260, NCT01837862, NCT02366884, NCT03925662, and NCT02201381) have tested its anti-cancer effects in combination with other drugs, which might suggest weak anti-cancer effects of MBZ as monotherapy and potential uses for synergizing effects with other drugs, as evidenced in the preclinical data.

Clinical evidence reveals that ABZ was administered at a dose of 10 mg/kg/day p.o., with two or three divided doses, in a pilot study [90]. In a phase 1 trial conducted on 36 patients, the maximum tolerated dose was 1200 mg twice daily (b.i.d.) p.o. (2400 mg/day) [89]. MBZ was administered at a dose of 100 mg b.i.d. p.o. in two case reports [91,92], while no exact maximal tolerated dose can be found in clinical trials, owing to the lack of reporting of results from these trials. All clinical evidence of benzimidazole drugs indicates that they were administered orally. ABZ has also been reported to be well tolerated in two studies [89,90]. Mainly, fatigue and mild gastrointestinal upset were reported [89]; however, in some patients, hematologic adverse events such as myelosuppression [89] or neutropenia [90] were also observed. In two case reports related to MBZ, each of which described one person, no significant adverse effects were described [91,92], but up to five-fold increases in levels of liver enzymes (aspartate aminotransferase and alanine aminotransferase) were detected in one patient [92]. Six clinical studies evaluating benzimidazole anthelmintics as anti-tumor agents are currently ongoing.

## 4. Pharmacokinetic Properties

There are limited pharmacokinetic data on the use of benzimidazole anthelmintics for humans, even with respect to ABZ, FBZ, FLB, MBZ, and OFZ (Table 3). It is well known that benzimidazoles are poorly soluble in water, which is the main reason for their low absorption and bioavailability [81,93,94,95]. In addition, dietary fat can substantially increase the absorption of benzimidazoles [81,95,96,97,98]. These five benzimidazoles are metabolized by first-pass metabolism [93,95,97,99].

However, details of the pharmacokinetic aspects differ depending on the benzimidazole. First, the metabolisms of ABZ and FBZ shared similar patterns, but that of MBZ is somewhat different. The main metabolic products, i.e., fenbendazole sulfoxide (FBZSO) and its sulfone derivative (FBZSO_2_), are produced upon first-pass metabolism of FBZ, while albendazole sulfoxide (ABZSO) and its sulfone derivative (ABZSO_2_) are produced through sequential oxidation upon first-pass metabolism of ABZ [99]. The metabolism of both ABZ and FBZ occurs by cytochrome P450 and flavin-monooxygenase [81], and the first metabolite of each, ABZSO and FBZSO, respectively, has two enantiomers in human plasma [99]. In contrast, MBZ is metabolized by extensive first-pass metabolism into many unidentified metabolites, and it is unclear which enzyme(s) carries this out [81]. In addition to metabolic pathways, metabolic rates also vary depending on each benzimidazole. ABZ is known to be metabolized by very rapid first-pass metabolism (T_1/2_ < 1.5 h) [100], compared to those of other benzimidazoles (T_1/2_ of FLZ in tissue, 1–2 d [95]; T_1/2_ of MBZ, 3–6 h [14]; and T_1/2_ of OFZ, 8.5–11 h [93]). In terms of excretion, ABZ, FBZ, MBZ, and their metabolites are eliminated in the feces and urine [81,96,99]. FLZ [95] and MBZ [96] have been reported to be mostly excreted in the feces. In addition, the excretion route of OFZ has not been clearly described [100].

The most important point that should be considered when judging whether benzimidazoles exert anti-cancer activities, as has been shown in a number of preclinical tests, is whether they can maintain effective concentrations consistently in the bloodstream of the human body. Based on several pharmacokinetic data, the maximum concentration (C_max_) of each benzimidazole was as follows: ABZ; 0.047–0.1 µM at a dose of 400 mg [100]; FLZ, 0.016 µM at a dose of 2 g [95]; MBZ, 0.47 µM at a dose of 10 mg/kg [97]; and OFZ, 21.5 µM at a dose of 60 mg/kg [93]. When the C_max_ of each benzimidazole was compared to the IC_50_ values obtained in a number of in vitro tests [14], the C_max_ values of ABZ and FLZ at those doses were regarded as lower, to exert effective anti-cancer effects on a variety of cancer cells, except for a small number of cells. Furthermore, since ABZ is rapidly metabolized into its main metabolites [94,96,101], the T_1/2_ of ABZ was observed to be less than 1.5 h [100]. This rapid metabolism may also be an obstacle to the use of this drug as an effective option for cancer treatment. The major challenge to repurposing benzimidazole anthelmintics as anti-cancer medicines will likely be improving their bioavailability by developing new formulations with better solubility, absorption, and longer half-life in order to achieve effective concentrations for enough time.

## 5. Discussion

Microtubule disruption has been a target for cancer treatment for a long time [18]. Because benzimidazole anthelmintics exert an anti-parasitic effect by disrupting microtubule polymerization by binding to β-tubulin [19,20], benzimidazoles might have anti-proliferative effects on cancer cells. Indeed, the anti-cancer and anti-growth effects of benzimidazoles were observed serendipitously upon their use as anti-parasitics during tests in animals [20,50,74], with the anti-cancer efficacy of the benzimidazole group demonstrated in a number of in vitro and in vivo studies. Furthermore, its predominant cancer-suppressing activities compared to those of other compounds have also been demonstrated in screening tests [39,51,54,67,71,73,86]. The anti-cancer mechanisms of benzimidazoles have not been clearly elucidated, but multiple mechanisms [10] have been identified which might contribute to their cancer-suppressing effects. As shown in Figure 1, the effects are mainly mediated through the disruption of microtubule polymerization [21,22,23,24,25,26], induction of apoptosis [19,26,27,28,29,30,31], or inhibition of angiogenesis [32,33] and metastasis [19,26,33,34], and as recently reported, autophagy induction [19,56,57,60,102], glycolysis suppression [32,52], immune system modulation [103], and cancer stemness inhibition [104]. In addition, it was revealed that this chemical group displays advantages in suppressing hard-to-treat cancers such as TNBC [19,28,35,55,57,66], brain cancer [20,44,50,54,62,63,64,65,74,75], and KRAS-mutant lung cancer [39], but also chemo-resistant cancer cells [58,59,69,70] in preclinical studies, with the possibility of synergizing with established conventional therapies, including radiation [26,39,42,43,59,60,73,88]. Therefore, the expectations of repurposing this drug group as a cancer treatment have increased in recent years.

However, despite these positive results, there is limited clinical evidence. One phase 1 clinical trial [89] and one pilot study [90] reported modest anti-tumor effects of ABZ, including a reduction in the levels of tumor markers. In two case reports, metastases regressed after treatment with MBZ in adrenal cancer [91] and metastatic colon cancer [92], while adrenocortical carcinoma did not progress for 19 months during the application of MBZ [91]. No studies have reported anti-tumor effects of these compounds in a large population.

We attempted to understand why the aforementioned anti-cancer effects were not observable in actual clinical settings. First, after reviewing a variety of preclinical studies, we determined that even though many factors and pathways related to the anti-cancer effects of benzimidazoles have been identified, the comprehensive mechanisms or the exact main target(s) resulting in these anti-cancer activities have not been completely clarified. Therefore, drug development for repurposing benzimidazoles as a cancer treatment inevitably faces certain uncertainties at present, such as difficulties in patient selection during clinical trials. To date, clinical trials related to the application of benzimidazole for cancer treatment have mostly recruited participants with brain cancer (NCT01729260, NCT02644291, and NCT01837862), colon cancer (NCT03925662), or solid tumors without detailed classifications ([89], NCT00003709, NCT02366884, and NCT02201381). The criteria for patient selection did not include any specific information such as cancer cell line types or genetic characteristics of the subject patients. Clarification of the precise anti-cancer mechanism and the main targets would help narrow down the range of subjects for participants.

Second, we tried to understand the nature of the anti-cancer effects of benzimidazoles in order to clarify the low efficacies observed in clinical evidence, and identified several factors. Based on the preclinical results, the efficacies of benzimidazoles were revealed to be very different, depending on the different cancer cell lines and benzimidazole types used. Specifically, in three screening studies [39,51,86] that tested against melanoma (M-14 and SK-Mel-19), K-RAS-mutant lung cancer (A-549, H-23, and H-1573), and colon cancer (HCT116 and RKO) cell lines, several benzimidazoles showed inhibitory effects, but the levels of these effects differed depending on the benzimidazole type. The benzimidazoles that exerted the most effective suppression were MBZ against melanoma, MTZ and FBZ against K-RAS-mutant lung cancer, and OBZ and MBZ against colon cancer. In a study conducted by Králová et al., similar results were observed. ABZ and FLZ exhibited very high inhibition of colon cancer cell lines (SW480, SW620, Caco2, and HCT8), whereas RBZ was ineffective [88]. Thus, a specific benzimidazole does not seem to have a predominant anti-cancer effect. Moreover, we found evidence of the anti-cancer effects of benzimidazoles in an extensive study conducted by Michaelis et al. [54]. In this study, researchers tested the inhibitory effects of FLZ on 321 cancer cell lines with 26 cancer entities. They found that FLZ had an IC_90_ of less than 5 µM for all 26 cancer entities. Above all, three entities, i.e., myeloma, neuroblastoma, and leukemia, showed high sensitivity to FLZ, with a mean IC_90_ of less than 1 µM, which was demonstrated to be achievable in mice, whereas only 117 (36%) of the total 321 cell lines had an IC_90_ of less than 1 µM. Based on this, we determined that the anti-cancer effects of FLZ also depended on cancer entities. Cell line dependency has also been reported for the anti-cancer effects of paclitaxel, in which the mechanism of cytotoxicity was revealed to be upregulating death receptor 5, thereby activating the extrinsic pathway of apoptosis in prostate cancer cell lines, but not in NSCLC or breast cancer cell lines [48]. These results indicate that differences in intracellular signal transduction pathways between cell lines may cause cell line dependency. To confirm these observations and extend this idea to the anti-cancer effects of other benzimidazoles, the extensive screening of other benzimidazoles should be performed in the near future.

This variation in the anti-cancer effects of benzimidazoles on the basis of the type of cancer entity, cell line, or benzimidazole type, affects the results of the clinical trials. Because of this, benzimidazoles might have limited suppressive effects on extensive cancer entities or even a cancer entity with various cell lines, in clinical trials, unless their concentrations in the bloodstream are sufficiently increased to inhibit a wide range of cancer cells. Although various benzimidazoles have shown anti-tumor activities in many preclinical studies, a sufficient level of efficacy should be demonstrated in a large number of participants, through trials, for them to be developed as an anti-cancer therapy. Therefore, when planning a clinical trial, the experimental group should be specifically restricted to participants with a specific cancer type demonstrated to be susceptible to the subject benzimidazole, or alternatively, a benzimidazole type that has already revealed its anti-cancer effects at relatively low concentrations could be selected; in either case, conducting trials on a large scale is a good development strategy to help increase the effectiveness of the approach. To acquire information for the determination of the abovementioned matters, more extensive preclinical data should be collected for benzimidazoles.

Finally, the last obstacle to understanding the anti-cancer effects of benzimidazoles is their low bioavailability. As explained, the C_max_ values for ABZ (0.047–0.1 µM at a one-time dose of 400 mg [100]) and FLZ (0.016 µM at a one-time dose of 2 g [95]) were considered to be low compared to the IC_50_ values observed in various in vitro tests [14]. In a pilot study and a phase 1 trial that reported the anti-cancer effects of ABZ, it was administered at a dose of more than 400 mg, that is, 10 mg/kg/day with two or three divided doses p.o. [90] and 400–1200 mg b.i.d. p.o. [89], respectively. As such, the poor solubility and absorption of benzimidazoles [81,93,94,95] or the rapid metabolism of ABZ [94,96,101] can also be attributed to their low anti-cancer efficacy in clinical settings. Therefore, there is a need to use different excipients or novel formulation technologies to increase the solubility and absorption of these compounds. In addition, benzimidazoles were administered orally in all the clinical trials described in Table 2; however, administration by injection could also be considered for better efficacy, considering that in most in vivo tests, other than those for MBZ, drugs were not administered orally.

## 6. Conclusions

Taken together, although the anthelmintics of the benzimidazole group have shown anti-cancer effects in many in vitro and in vivo studies, there is still limited clinical evidence for their anti-cancer effects. Moreover, modest efficacy has been observed for ABZ and MBZ. We presume that this can be explained by fact that the main targets of these drugs and their multiple anti-cancer properties are poorly understood, as well as the low bioavailability of these compounds. Therefore, future studies should investigate novel formulations and development strategies to enhance the anti-cancer effects of benzimidazoles in clinical applications.

## Figures and Tables

**Table 1:** Chemical Structures of Benzimidazoles.

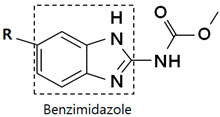			
R	Drug	R	Drug
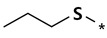	Albendazole	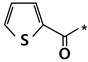	Nocodazole
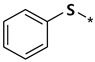	Fenbendazole	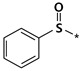	Oxfendazole
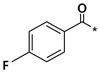	Flubendazole	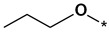	Oxibendazole
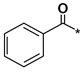	Mebendazole	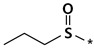	Ricobendazole
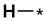	Carbendazim	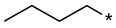	Parbendazole
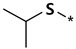	Methiazole		

**Table 2:** Clinical Evidence of Benzimidazoles for Anti-tumor Effect.

Drug	Stage	Cancer Type	Number of Patients	Methods	Adverse Effects	Results	Identifier/Ref.
Albendazole	Phase 1	Refractory solid tumors	36	Every day for 2 weeks, followed by 1 week of rest. Treatment was repeated in a 21-day cycle. 400–1200 mg b.i.d. p.o.	ABZ was well tolerated. Fatigue and mild gastrointestinal upset (Major). Myelosuppression.	16% of patients showed a decrease in levels of tumor markers. Plasma VEGF level decreased in the first 8 h after ABZ administration.	[89]
Albendazole	Pilot Study	Colorectal cancer or hepatocellular carcinoma	7	10 mg/kg/day, with 2 or 3 divided doses p.o. (28 d). The maximum tolerated dose was 1200 mg b.i.d.	ABZ was well tolerated. Severe neutropenia in three patients.	CEA decreased in two patients. CEA or α-feto protein stabilized in three patients.	[90]
Carbendazim	Phase 1	Unspecified adult solid tumor	25	P.o weekly for 3 consecutive weeks, followed by 1 week of rest. Treatment repeated in a 28-day cycle. Determining dose.	No results posted.	No results posted. Actual study completion date: November 2000	NCT00003709
Mebendazole	Case report	Adrenal cancer	1	100 mg b.i.d. p.o. for 19 months.	No significant adverse effects.	Metastases regressed. The patient’s disease remained stable for 19 months, but showed progression after 24 months.	[91]
Mebendazole	Case report	Refractory metastatic colon cancer	1	100 mg b.i.d. p.o. for six weeks.	AST and ALT were increased up to > five times above the normal limit.	The metastases in the lungs and lymph nodes were near completely remissioned. A good portion of those in the liver were remissioned.	[92]
Mebendazole	Not applicable	Advanced hepatocellular carcinoma	170 (recruiting)	100 mg b.i.d. p.o. in combination with lenvatinib.	No results posted.	No results posted. Estimated study completion date: 19 June 2022	NCT04443049
Mebendazole	Phase 1	High-grade glioma	24	T.i.d. p.o. in a 28-day cycle, in combination with temozolomide. Determining dose.	No results posted.	No results posted. Actual study completion date: 16 April 2021	NCT01729260
Mebendazole	Phase 1	Recurrent pediatric brain cancers	21 (recruiting)	T.i.d. p.o. Determining dose.	No results posted.	No results posted. Estimated study completion date: June 2022	NCT02644291
Mebendazole	Phase 1/2	Pediatric gliomas	36 (recruiting)	50–200 mg/kg/day divided twice p.o., in combination with standard anti-tumor drugs	No results posted.	No results posted. Estimated study completion date: April 2023	NCT01837862
Mebendazole	Phase 2a	Advanced gastrointestinal cancer or cancer of unknown origin	11 (Terminated due to lack of effect)	50–4000 mg b.i.d. p.o. for 16 weeks. Determining dose.	No results posted.	No results posted. Actual study completion date: 16 January 2019	NCT03628079
Mebendazole	Phase 2	Incurable and lethal cancers	250 (recruiting)	Tolerable and safe doses for 10 to 12 months. Combination of two anti-protozoal drugs.	No results posted.	No results posted. Estimated study completion date: 31 December 2023	NCT02366884
Mebendazole	Phase 3	Colorectal cancer	40 (recruiting)	Folfox with avastin and MBZ.	No results posted.	No results posted. Estimated study completion date: December 2028	NCT03925662
Mebendazole	Phase 3	Cancer	207 (Not yet recruiting)	100 mg q.d. in combination with atorvastatin, metformin, and doxycycline.	No results posted.	No results posted. Estimated study completion date: 22 September 2026	NCT02201381

Abbreviations: b.i.d: twice daily; p.o.: oral administration; ABZ: albendazole; VEGF: vascular endothelial growth factor; CEA: carcinoembryonic antigen; AST: aspartate aminotransferase; ALT: alanine aminotransferase; t.i.d.: three times a day; q.d.: once daily.

**Table 3:** Pharmacokinetic Properties of Benzimidazoles.

Drug	Absorption	Distribution	Metabolism	Excretion	Ref.
Albendazole	· <5% · Poor solubility, as well as low absorption and bioavailability. · High inter-variabilities of peak levels. · A dose of 400 mg p.o. led to a C_max_ of 0.16–1.58 mg/L for ABZSO. · T_max_ of ABZ was <2–3 h. · Fat in the diet increased the absorption up to 6.5-fold. · T_max_ of ABZSO was 4.75 h. · C_max_ of ABZSO was 1.20 ± 0.44 μg/mL. · C_max_ of ABZ was 12.5 [0.047 µM] to 26.5 ng/mL [0.1 µM].	· ABZSO was widely distributed. About 70% of ABZSO was bound to plasma proteins, whereas about 90% of ABZ was bound to them. · ABZSO crossed the BBB. · ABZSO enantiomers were distributed about two-fold higher in the plasma than in the cerebrospinal fluid, in humans. · When treated with 400 mg ABZ, a small amount of ABZ was detected in the serum from 2–8 h after administration. · ABZSO was detected until 72 h in the blood.	· ABZ is metabolized to ABZSO by very rapid first-pass metabolism, and finally to ABZ sulfone through further conversion. · Metabolism is carried out by cytochrome P450 and other oxidases, including flavin-monooxygenase. · ABZSO has two enantiomers in the human plasma. (+)-ABZSO is the predominant enantiomeric form in the human plasma. · Increased CYP1A expression can cause auto-inductive effect of ABZ, upon repeated administration of ABZ.	· T_1/2_ of ABZSO is 8–14 h. · T_1/2_ of ABZ is <1.5 h. · ABZ and its metabolites are excreted in the urine and feces. · ABZSO is excreted in the urine quickly, from 4–72 h after administration. · ABZ concentrations are too low to measure in the urine.	[81,94,96,99,100,101]
Fenbendazole	-	-	· FBZ is metabolized to FBZSO by first-pass metabolism, and finally to FBZ sulfone by means of further conversion. · Metabolism is carried out by cytochrome P450 and flavin-monooxygenase. · FBZSO has two enantiomers in the human plasma.	· FBZ and its metabolites are excreted in the urine and feces.	[99]
Flubendazole	· Poor solubility as well as low absorption and bioavailability. · A dose of 2 g p.o. led to a C_max_ that was lower than 5 ng/mL [0.016 µM] for FLZ. · Administration after a meal increases absorption.	-	· Initial biotransformation takes place through first-pass metabolism.	· FLZ is excreted in the feces (more than 80%) and urine. · T_1/2_ in tissues is 1–2 d.	[95]
Mebendazole	· 5–10% and 17–22% · poor solubility. · Fat in the diet increased the absorption more than 5-fold. · C_max_ of MBZ was 137.4 ng/mL [0.47 µM], at a dose of 10 mg/kg. · T_max_ of MBZ was 2–4 h. · High inter-variabilities of peak levels.	· 90–95% of it existed as bound to plasma proteins.	· MBZ is metabolized by extensive first-pass metabolism to many unidentified metabolites. · It is unclear which enzymes carry out this metabolism.	· MBZ and its metabolites are excreted in the feces and urine. · T_1/2_ is 3–6 h.	[14,81,96,97]
Oxfendazole	· Poor solubility, but higher than that of ABZ or FBZ. · C_max_ of OFZ was 6770 ng/mL [21.5 µM], at a dose of 60 mg/kg. · T_max_ of OFZ was 2–3 h. · Fat in the diet increased the C_max_ by 49%, and AUC by 86%.	-	· OFZ is metabolized to OFZ sulfone, FBZ, OFZ sulfate conjugates, and OFZ glucuronide conjugates.	· Minimal amount (<1% of dose) of OFZ is excreted in the urine. · T_1/2_ is 8.5–11 h.	[93,98]

Abbreviations: ABZ: albendazole; ABZSO: ABZ sulfoxide; T_max_: time to peak drug concentration; C_max_: maximum concentration; BBB: blood-brain barrier; FBZ: fenbendazole; FBZSO: FBZ sulfoxide; p.o.: oral administration; FLZ: flubendazole; T_1/2_: half-life time; MBZ: mebendazole; OFZ: oxfendazole; AUC: area under the concentration-time curve.

## References

[1] Harris P., Finlay I.G., Cook A., Thomas K.J., Hood K. (2003). Complementary and alternative medicine use by patients with cancer in Wales: A cross sectional survey. Complement. Ther. Med..

[2] Kufel-Grabowska J., Bartoszkiewicz M., Litwiniuk M. (2021). The use of complementary and alternative medicine among cancer patients. Polskie Arch. Med. Wewnetrznej.

[3] Eguchi K., Hyodo I., Saeki H. (2000). Current status of cancer patients’ perception of alternative medicine in Japan. Support Care Cancer.

[4] Jung K.-W., Won Y.-J., Kong H.-J., Lee E.S. (2019). Cancer statistics in Korea: Incidence, mortality, survival, and prevalence in 2016. Cancer Res. Treat..

[5] American Cancer Society (2022). Cancer Facts & Figures 2022.

[6] Sung H., Ferlay J., Siegel R.L., Laversanne M., Soerjomataram I., Jemal A., Bray F. (2021). Global cancer statistics 2020: GLOBOCAN estimates of incidence and mortality worldwide for 36 cancers in 185 countries. CA Cancer J. Clin..

[7] Finn R.S., Qin S., Ikeda M., Galle P.R., Ducreux M., Kim T.-Y., Kudo M., Breder V., Merle P., Kaseb A.O. (2020). Atezolizumab plus bevacizumab in unresectable hepatocellular carcinoma. N. Engl. J. Med..

[8] Debela D.T., Muzazu S.G., Heraro K.D., Ndalama M.T., Mesele B.W., Haile D.C., Kitui S.K., Manyazewal T. (2021). New approaches and procedures for cancer treatment: Current perspectives. SAGE Open Med..

[9] Abou-Gharbia M., Childers W.E. (2014). Discovery of innovative therapeutics: Today’s realities and tomorrow’s vision. 2. Pharma’s challenges and their commitment to innovation. J. Med. Chem..

[10] Nath J., Paul R., Ghosh S.K., Paul J., Singha B., Debnath N. (2020). Drug repurposing and relabeling for cancer therapy: Emerging benzimidazole antihelminthics with potent anticancer effects. Life Sci..

[11] Wouters O.J., McKee M., Luyten J. (2020). Estimated research and development investment needed to bring a new medicine to market, 2009–2018. JAMA.

[12] Iragorri N., de Oliveira C., Fitzgerald N., Essue B. (2021). The Out-of-Pocket Cost Burden of Cancer Care—A Systematic Literature Review. Curr. Oncol..

[13] Zhang Z., Zhou L., Xie N., Nice E.C., Zhang T., Cui Y., Huang C. (2020). Overcoming cancer therapeutic bottleneck by drug repurposing. Signal Transduct. Target. Ther..

[14] Son D.-S., Lee E.-S., Adunyah S.E. (2020). The antitumor potentials of benzimidazole anthelmintics as repurposing drugs. Immune Netw..

[15] Kim E.-Y. Dog Dewormer Goes Out of Stock Amid Rumor of Efficacy for Cancer. *Korea Biomedical Review*, 27 December 2019. https://www.koreabiomed.com/news/articleView.html?idxno=7073.

[16] Laudisi F., Marônek M., Di Grazia A., Monteleone G., Stolfi C. (2020). Repositioning of anthelmintic drugs for the treatment of cancers of the digestive system. Int. J. Mol. Sci..

[17] U.S. Food & Drug Administration Drugs@FDA: FDA-Approved Drugs. https://www.accessdata.fda.gov/scripts/cder/daf/index.cfm.

[18] Markowitz D., Ha G., Ruggieri R., Symons M. (2017). Microtubule-targeting agents can sensitize cancer cells to ionizing radiation by an interphase-based mechanism. OncoTargets Ther..

[19] Liu H., Sun H., Zhang B., Liu S., Deng S., Weng Z., Zuo B., Yang J., He Y. (2020). 18 F-FDG PET imaging for monitoring the early anti-tumor effect of albendazole on triple-negative breast cancer. Breast Cancer.

[20] Bai R.-Y., Staedtke V., Aprhys C.M., Gallia G.L., Riggins G.J. (2011). Antiparasitic mebendazole shows survival benefit in 2 preclinical models of glioblastoma multiforme. Neuro Oncol..

[21] Zhang X., Zhao J., Gao X., Pei D., Gao C. (2017). Anthelmintic drug albendazole arrests human gastric cancer cells at the mitotic phase and induces apoptosis. Exp. Ther. Med..

[22] Mrkvová Z., Uldrijan S., Pombinho A., Bartůněk P., Slaninová I. (2019). Benzimidazoles downregulate Mdm2 and MdmX and activate p53 in MdmX overexpressing tumor cells. Molecules.

[23] Čáňová K., Rozkydalová L., Vokurková D., Rudolf E. (2018). Flubendazole induces mitotic catastrophe and apoptosis in melanoma cells. Toxicol. Vitr..

[24] Sasaki J.-I., Ramesh R., Chada S., Gomyo Y., Roth J.A., Mukhopadhyay T. (2002). The Anthelmintic Drug Mebendazole Induces Mitotic Arrest and Apoptosis by Depolymerizing Tubulin in Non-Small Cell Lung Cancer Cells 1 Supported in part by grants from the National Cancer Institute and the NIH Specialized Program of Research Excellence in Lung Cancer P-50-CA70907 and P01 CA78778-01A1 (both to JAR), by gifts to the Division of Surgery and Anesthesiology from Tenneco and Exxon for the Core Laboratory Facility, by The University of Texas MD Anderson Cancer Center Support Core Grant CA16672, by the WM Keck Foundation, and by a sponsored research agreement with Introgen Therapeutics, Inc. JAR is a scientific advisor for Introgen Therapeutics, Inc. 1. Mol. Cancer Ther..

[25] Yenjerla M., Cox C., Wilson L., Jordan M.A. (2009). Carbendazim inhibits cancer cell proliferation by suppressing microtubule dynamics. J. Pharmacol. Exp. Ther..

[26] Florio R., Veschi S., di Giacomo V., Pagotto S., Carradori S., Verginelli F., Cirilli R., Casulli A., Grassadonia A., Tinari N. (2019). The benzimidazole-based anthelmintic parbendazole: A repurposed drug candidate that synergizes with gemcitabine in pancreatic cancer. Cancers.

[27] Dogra N., Mukhopadhyay T. (2012). Impairment of the ubiquitin-proteasome pathway by methyl N-(6-phenylsulfanyl-1H-benzimidazol-2-yl) carbamate leads to a potent cytotoxic effect in tumor cells: A novel antiproliferative agent with a potential therapeutic implication. J. Biol. Chem..

[28] Oh E., Kim Y.J., An H., Sung D., Cho T.M., Farrand L., Jang S., Seo J.H., Kim J.Y. (2018). Flubendazole elicits anti-metastatic effects in triple-negative breast cancer via STAT3 inhibition. Int. J. Cancer.

[29] Pinto L.C., Mesquita F.P., Soares B.M., da Silva E.L., Puty B., de Oliveira E.H.C., Burbano R.R., Montenegro R.C. (2019). Mebendazole induces apoptosis via C-MYC inactivation in malignant ascites cell line (AGP01). Toxicol. In Vitro.

[30] Wei K.-L., Chen F.-Y., Lin C.-Y., Gao G.-L., Kao W.-Y., Yeh C.-H., Chen C.-R., Huang H.-C., Tsai W.-R., Jong K.-J. (2016). Activation of aryl hydrocarbon receptor reduces carbendazim-induced cell death. Toxicol. Appl. Pharmacol..

[31] Chen Q., Li Y., Zhou X., Li R. (2018). Oxibendazole inhibits prostate cancer cell growth. Oncol. Lett..

[32] Zhou F., Du J., Wang J. (2017). Albendazole inhibits HIF-1α-dependent glycolysis and VEGF expression in non-small cell lung cancer cells. Mol. Cell. Biochem..

[33] Sung S.J., Kim H.-K., Hong Y.-K., Joe Y.A. (2019). Autophagy is a potential target for enhancing the anti-angiogenic effect of mebendazole in endothelial cells. Biomol. Ther..

[34] Kralova V., Hanušová V., Caltová K., Špaček P., Hochmalová M., Skálová L., Rudolf E. (2018). Flubendazole and mebendazole impair migration and epithelial to mesenchymal transition in oral cell lines. Chem. Biol. Interact..

[35] Priotti J., Baglioni M.V., García A., Rico M.J., Leonardi D., Lamas M.C., Márquez M.M. (2018). Repositioning of anti-parasitic drugs in cyclodextrin inclusion complexes for treatment of triple-negative breast cancer. AAPS PharmSciTech.

[36] Pourgholami M.H., Akhter J., Wang L., Lu Y., Morris D.L. (2005). Antitumor activity of albendazole against the human colorectal cancer cell line HT-29: In vitro and in a xenograft model of peritoneal carcinomatosis. Cancer Chemother. Pharmacol..

[37] Wang L.-J., Lee Y.-C., Huang C.-H., Shi Y.-J., Chen Y.-J., Pei S.-N., Chou Y.-W., Chang L.-S. (2019). Non-mitotic effect of albendazole triggers apoptosis of human leukemia cells via SIRT3/ROS/p38 MAPK/TTP axis-mediated TNF-α upregulation. Biochem. Pharmacol..

[38] Pourgholami M., Woon L., Almajd R., Akhter J., Bowery P., Morris D. (2001). In vitro and in vivo suppression of growth of hepatocellular carcinoma cells by albendazole. Cancer Lett..

[39] Shimomura I., Yokoi A., Kohama I., Kumazaki M., Tada Y., Tatsumi K., Ochiya T., Yamamoto Y. (2019). Drug library screen reveals benzimidazole derivatives as selective cytotoxic agents for KRAS-mutant lung cancer. Cancer Lett..

[40] Chu S.W., Badar S., Morris D.L., Pourgholami M.H. (2009). Potent inhibition of tubulin polymerisation and proliferation of paclitaxel-resistant 1A9PTX22 human ovarian cancer cells by albendazole. Anticancer Res..

[41] Pourgholami M.H., Cai Z.Y., Lu Y., Wang L., Morris D.L. (2006). Albendazole: A potent inhibitor of vascular endothelial growth factor and malignant ascites formation in OVCAR-3 tumor-bearing nude mice. Clin. Cancer Res..

[42] Patel K., Doudican N.A., Schiff P.B., Orlow S.J. (2011). Albendazole sensitizes cancer cells to ionizing radiation. Radiat. Oncol..

[43] Patel K., Doudican N., Schiff P., Orlow S. (2011). Albendazole Sensitizes Melanoma and Small Cell Lung Cancer Cells to Ionizing Radiation. Int. J. Radiat. Oncol. Biol. Phys..

[44] Tang Y., Liang J., Wu A., Chen Y., Zhao P., Lin T., Zhang M., Xu Q., Wang J., Huang Y. (2017). Co-delivery of trichosanthin and albendazole by nano-self-assembly for overcoming tumor multidrug-resistance and metastasis. ACS Appl. Mater. Interfaces.

[45] Noorani L., Stenzel M., Liang R., Pourgholami M.H., Morris D.L. (2015). Albumin nanoparticles increase the anticancer efficacy of albendazole in ovarian cancer xenograft model. J. Nanobiotechnol..

[46] Choi E.-K., Kim S.-W., Nam E.-J., Paek J., Yim G.-W., Kang M.-H., Kim Y.-T. (2011). Differential effect of intraperitoneal albendazole and paclitaxel on ascites formation and expression of vascular endothelial growth factor in ovarian cancer cell-bearing athymic nude mice. Reprod. Sci..

[47] Hettiarachchi G., Samanta S.K., Falcinelli S., Zhang B., Moncelet D., Isaacs L., Briken V. (2016). Acyclic cucurbit [n] uril-type molecular container enables systemic delivery of effective doses of albendazole for treatment of SK-OV-3 xenograft tumors. Mol. Pharm..

[48] Ehteda A., Galettis P., Pillai K., Morris D.L. (2013). Combination of albendazole and 2-methoxyestradiol significantly improves the survival of HCT-116 tumor-bearing nude mice. BMC Cancer.

[49] Ehteda A., Galettis P., Chu S.W.L., Pillai K., Morris D.L. (2012). Complexation of albendazole with hydroxypropyl-β-cyclodextrin significantly improves its pharmacokinetic profile, cell cytotoxicity and antitumor efficacy in nude mice. Anticancer Res..

[50] Lai S.R., Castello S., Robinson A., Koehler J. (2017). In vitro anti-tubulin effects of mebendazole and fenbendazole on canine glioma cells. Vet. Comp. Oncol..

[51] Nygren P., Fryknäs M., Ågerup B., Larsson R. (2013). Repositioning of the anthelmintic drug mebendazole for the treatment for colon cancer. J. Cancer Res. Clin. Oncol..

[52] Dogra N., Kumar A., Mukhopadhyay T. (2018). Fenbendazole acts as a moderate microtubule destabilizing agent and causes cancer cell death by modulating multiple cellular pathways. Sci. Rep..

[53] Duan Q., Liu Y., Rockwell S. (2013). Fenbendazole as a potential anticancer drug. Anticancer Res..

[54] Michaelis M., Agha B., Rothweiler F., Löschmann N., Voges Y., Mittelbronn M., Starzetz T., Harter P.N., Abhari B.A., Fulda S. (2015). Identification of flubendazole as potential anti-neuroblastoma compound in a large cell line screen. Sci. Rep..

[55] Hou Z.-J., Luo X., Zhang W., Peng F., Cui B., Wu S.-J., Zheng F.-M., Xu J., Xu L.-Z., Long Z.-J. (2015). Flubendazole, FDA-approved anthelmintic, targets breast cancer stem-like cells. Oncotarget.

[56] Zhang L., Guo M., Li J., Zheng Y., Zhang S., Xie T., Liu B. (2015). Systems biology-based discovery of a potential Atg4B agonist (Flubendazole) that induces autophagy in breast cancer. Mol. Biosyst..

[57] Zhen Y., Zhao R., Wang M., Jiang X., Gao F., Fu L., Zhang L., Zhou X.-L. (2020). Flubendazole elicits anti-cancer effects via targeting EVA1A-modulated autophagy and apoptosis in Triple-negative Breast Cancer. Theranostics.

[58] Kim Y.-J., Sung D., Oh E., Cho Y., Cho T.-M., Farrand L., Seo J.H., Kim J.Y. (2018). Flubendazole overcomes trastuzumab resistance by targeting cancer stem-like properties and HER2 signaling in HER2-positive breast cancer. Cancer Lett..

[59] Spagnuolo P.A., Hu J., Hurren R., Wang X., Gronda M., Sukhai M.A., Di Meo A., Boss J., Ashali I., Beheshti Zavareh R. (2010). The antihelmintic flubendazole inhibits microtubule function through a mechanism distinct from Vinca alkaloids and displays preclinical activity in leukemia and myeloma. Blood.

[60] Lin S., Yang L., Yao Y., Xu L., Xiang Y., Zhao H., Wang L., Zuo Z., Huang X., Zhao C. (2019). Flubendazole demonstrates valid antitumor effects by inhibiting STAT3 and activating autophagy. J. Exp. Clin. Cancer Res..

[61] Li Y., Acharya G., Elahy M., Xin H., Khachigian L.M. (2019). The anthelmintic flubendazole blocks human melanoma growth and metastasis and suppresses programmed cell death protein-1 and myeloid-derived suppressor cell accumulation. Cancer Lett..

[62] Kipper F.C., Silva A.O., Marc A.L., Confortin G., Junqueira A.V., Neto E.P., Lenz G. (2018). Vinblastine and antihelmintic mebendazole potentiate temozolomide in resistant gliomas. Investig. New Drugs.

[63] De Witt M., Gamble A., Hanson D., Markowitz D., Powell C., Al Dimassi S., Atlas M., Boockvar J., Ruggieri R., Symons M. (2017). Repurposing mebendazole as a replacement for vincristine for the treatment of brain tumors. Mol. Med..

[64] Skibinski C.G., Williamson T., Riggins G.J. (2018). Mebendazole and radiation in combination increase survival through anticancer mechanisms in an intracranial rodent model of malignant meningioma. J. Neurooncol..

[65] Bai R.-Y., Staedtke V., Wanjiku T., Rudek M.A., Joshi A., Gallia G.L., Riggins G.J. (2015). Brain penetration and efficacy of different mebendazole polymorphs in a mouse brain tumor model. Clin. Cancer Res..

[66] Zhang L., Dratver M.B., Yazal T., Dong K., Nguyen A., Yu G., Dao A., Dratver M.B., Duhachek-Muggy S., Bhat K. (2019). Mebendazole potentiates radiation therapy in triple-negative breast cancer. Int. J. Radiat. Oncol. Biol. Phys..

[67] He L., Shi L., Du Z., Huang H., Gong R., Ma L., Chen L., Gao S., Lyu J., Gu H. (2018). Mebendazole exhibits potent anti-leukemia activity on acute myeloid leukemia. Exp. Cell Res..

[68] Zhang F., Li Y., Zhang H., Huang E., Gao L., Luo W., Wei Q., Fan J., Song D., Liao J. (2017). Anthelmintic mebendazole enhances cisplatin’s effect on suppressing cell proliferation and promotes differentiation of head and neck squamous cell carcinoma (HNSCC). Oncotarget.

[69] Pinto L.C., Moreira-Nunes C.d.F.A., Soares B.M., Burbano R.M.R., de Lemos J.A.R., Montenegro R.C. (2017). Mebendazole, an antiparasitic drug, inhibits drug transporters expression in preclinical model of gastric peritoneal carcinomatosis. Toxicol. Vitr..

[70] Wang X., Lou K., Song X., Ma H., Zhou X., Xu H., Wang W. (2020). Mebendazole is a potent inhibitor to chemoresistant T cell acute lymphoblastic leukemia cells. Toxicol. Appl. Pharmacol..

[71] Tan Z., Chen L., Zhang S. (2016). Comprehensive modeling and discovery of mebendazole as a novel TRAF2-and NCK-interacting kinase inhibitor. Sci. Rep..

[72] Li Y., Thomas D., Deutzmann A., Majeti R., Felsher D.W., Dill D.L. (2019). Mebendazole for differentiation therapy of acute myeloid leukemia identified by a lineage maturation index. Sci. Rep..

[73] Simbulan-Rosenthal C.M., Dakshanamurthy S., Gaur A., Chen Y.-S., Fang H.-B., Abdussamad M., Zhou H., Zapas J., Calvert V., Petricoin E.F. (2017). The repurposed anthelmintic mebendazole in combination with trametinib suppresses refractory NRASQ61K melanoma. Oncotarget.

[74] Larsen A.R., Bai R.-Y., Chung J.H., Borodovsky A., Rudin C.M., Riggins G.J., Bunz F. (2015). Repurposing the antihelmintic mebendazole as a hedgehog inhibitor. Mol. Cancer Ther..

[75] Bai R.-Y., Staedtke V., Rudin C.M., Bunz F., Riggins G.J. (2015). Effective treatment of diverse medulloblastoma models with mebendazole and its impact on tumor angiogenesis. Neuro Oncol..

[76] Younis N.S., Ghanim A.M., Saber S. (2019). Mebendazole augments sensitivity to sorafenib by targeting MAPK and BCL-2 signalling in n-nitrosodiethylamine-induced murine hepatocellular carcinoma. Sci. Rep..

[77] Williamson T., Bai R.-Y., Staedtke V., Huso D., Riggins G.J. (2016). Mebendazole and a non-steroidal anti-inflammatory combine to reduce tumor initiation in a colon cancer preclinical model. Oncotarget.

[78] Doudican N.A., Byron S.A., Pollock P.M., Orlow S.J. (2013). XIAP downregulation accompanies mebendazole growth inhibition in melanoma xenografts. Anticancer Drugs.

[79] Martarelli D., Pompei P., Baldi C., Mazzoni G. (2008). Mebendazole inhibits growth of human adrenocortical carcinoma cell lines implanted in nude mice. Cancer Chemother. Pharmacol..

[80] Rushworth L.K., Hewit K., Munnings-Tomes S., Somani S., James D., Shanks E., Dufès C., Straube A., Patel R., Leung H.Y. (2020). Repurposing screen identifies mebendazole as a clinical candidate to synergise with docetaxel for prostate cancer treatment. Br. J. Cancer.

[81] Dayan A. (2003). Albendazole, mebendazole and praziquantel. Review of non-clinical toxicity and pharmacokinetics. Acta Trop..

[82] Atalay P.B., Kuku G., Tuna B.G. (2019). Effects of carbendazim and astaxanthin co-treatment on the proliferation of MCF-7 breast cancer cells. In Vitro Cell. Dev. Biol. Anim..

[83] Kawaratani Y., Matsuoka T., Hirata Y., Fukata N., Nagaoka Y., Uesato S. (2015). Influence of the carbamate fungicide benomyl on the gene expression and activity of aromatase in the human breast carcinoma cell line MCF-7. Environ. Toxicol. Pharmacol..

[84] Wales C.T., Taylor F.R., Higa A.T., McAllister H.A., Jacobs A.T. (2015). ERK-dependent phosphorylation of HSF1 mediates chemotherapeutic resistance to benzimidazole carbamates in colorectal cancer cells. Anticancer Drugs.

[85] Xu D., Tian W., Jiang C., Huang Z., Zheng S. (2019). The anthelmintic agent oxfendazole inhibits cell growth in non-small cell lung cancer by suppressing c-Src activation. Mol. Med. Rep..

[86] Doudican N., Rodriguez A., Osman I., Orlow S.J. (2008). Mebendazole induces apoptosis via Bcl-2 inactivation in chemoresistant melanoma cells. Mol. Cancer Res..

[87] Belaz K.R.A., Denadai M., Almeida A.P., Lima R.T., Vasconcelos M.H., Pinto M.M., Cass Q.B., Oliveira R.V. (2012). Enantiomeric resolution of albendazole sulfoxide by semipreparative HPLC and in vitro study of growth inhibitory effects on human cancer cell lines. J. Pharm. Biomed. Anal..

[88] Králová V., Hanušová V., Stanková P., Knoppová K., Cánová K., Skálová L. (2013). Antiproliferative effect of benzimidazole anthelmintics albendazole, ricobendazole, and flubendazole in intestinal cancer cell lines. Anticancer Drugs.

[89] Pourgholami M.H., Szwajcer M., Chin M., Liauw W., Seef J., Galettis P., Morris D.L., Links M. (2010). Phase I clinical trial to determine maximum tolerated dose of oral albendazole in patients with advanced cancer. Cancer Chemother. Pharmacol..

[90] Morris D.L., Jourdan J.-L., Pourgholami M.H. (2001). Pilot study of albendazole in patients with advanced malignancy. Oncology.

[91] Dobrosotskaya I.Y., Hammer G.D., Schteingart D.E., Maturen K.E., Worden F.P. (2011). Mebendazole monotherapy and long-term disease control in metastatic adrenocortical carcinoma. Endocr. Pract..

[92] Nygren P., Larsson R. (2014). Drug repositioning from bench to bedside: Tumour remission by the antihelmintic drug mebendazole in refractory metastatic colon cancer. Acta Oncol..

[93] An G., Murry D.J., Gajurel K., Bach T., Deye G., Stebounova L.V., Codd E.E., Horton J., Gonzalez A.E., Garcia H.H. (2019). Pharmacokinetics, safety, and tolerability of oxfendazole in healthy volunteers: A randomized, placebo-controlled first-in-human single-dose escalation study. Antimicrob. Agents Chemother..

[94] Jung-Cook H. (2012). Pharmacokinetic variability of anthelmintics: Implications for the treatment of neurocysticercosis. Expert Rev. Clin. Pharmacol..

[95] Čáňová K., Rozkydalová L., Rudolf E. (2017). Anthelmintic flubendazole and its potential use in anticancer therapy. Acta Med..

[96] Hong S.-T. (2018). Albendazole and praziquantel: Review and safety monitoring in Korea. Infect. Chemother..

[97] Pantziarka P., Bouche G., Meheus L., Sukhatme V., Sukhatme V.P. (2014). Repurposing Drugs in Oncology (ReDO)—Mebendazole as an anti-cancer agent. Ecancermedicalscience.

[98] Bach T., Galbiati S., Kennedy J.K., Deye G., Nomicos E.Y., Codd E.E., Garcia H.H., Horton J., Gilman R.H., Gonzalez A.E. (2020). Pharmacokinetics, safety, and tolerability of oxfendazole in healthy adults in an open-label phase 1 multiple ascending dose and food effect study. Antimicrob. Agents Chemother..

[99] Capece B.P., Virkel G.L., Lanusse C.E. (2009). Enantiomeric behaviour of albendazole and fenbendazole sulfoxides in domestic animals: Pharmacological implications. Vet. J..

[100] Schulz J.D., Neodo A., Coulibaly J.T., Keiser J. (2019). Pharmacokinetics of albendazole, albendazole sulfoxide, and albendazole sulfone determined from plasma, blood, dried-blood spots, and Mitra samples of hookworm-infected adolescents. Antimicrob. Agents Chemother..

[101] Ceballos L., Krolewiecki A., Juárez M., Moreno L., Schaer F., Alvarez L.I., Cimino R., Walson J., Lanusse C.E. (2018). Assessment of serum pharmacokinetics and urinary excretion of albendazole and its metabolites in human volunteers. PLoS Negl. Trop. Dis..

[102] Rudolf K., Rudolf E. (2020). An analysis of mitotic catastrophe induced cell responses in melanoma cells exposed to flubendazole. Toxicol. Vitr..

[103] Rubin J., Mansoori S., Blom K., Berglund M., Lenhammar L., Andersson C., Loskog A., Fryknäs M., Nygren P., Larsson R. (2018). Mebendazole stimulates CD14+ myeloid cells to enhance T-cell activation and tumour cell killing. Oncotarget.

[104] Zhang Q.-L., Lian D.-D., Zhu M.J., Li X.M., Lee J.K., Yoon T.-J., Lee J.-H., Jiang R.-H., Kim C.D. (2019). Antitumor effect of albendazole on cutaneous squamous cell carcinoma (SCC) cells. BioMed Res. Int..

